# Neuro-symbolic approaches in artificial intelligence

**DOI:** 10.1093/nsr/nwac035

**Published:** 2022-03-04

**Authors:** Pascal Hitzler, Aaron Eberhart, Monireh Ebrahimi, Md Kamruzzaman Sarker, Lu Zhou

**Affiliations:** Department of Computer Science, Kansas State University, USA; Department of Computer Science, Kansas State University, USA; Center for Open Source Data and AI Technologies (CODAIT), IBM Watson, USA; Department of Computing Sciences, University of Hartford, USA; Department of Computer Science, Kansas State University, USA

**Keywords:** artificial intelligence, deep learning, knowledge representation and reasoning, neuro-symbolic integration

Neuro-symbolic artificial intelligence refers to a field of research and applications that combines machine learning methods based on artificial neural networks, such as deep learning, with symbolic approaches to computing and artificial intelligence (AI), as can be found for example in the AI subfield of knowledge representation and reasoning. Neuro-symbolic AI has a long history; however, it remained a rather niche topic until recently, when landmark advances in machine learning—prompted by deep learning—caused a significant rise in interest and research activity in combining neural and symbolic methods. In this overview, we provide a rough guide to key research directions, and literature pointers for anybody interested in learning more about the field.

Neuro-symbolic artificial intelligence can be defined as the subfield of artificial intelligence (AI) that combines neural and symbolic approaches. By *neural* we mean approaches based on artificial neural networks—sometimes called connectionist or subsymbolic approaches—and in particular this includes deep learning, which has provided very significant breakthrough results in the recent decade, and is fueling the current general interest in AI. By *symbolic* we mean approaches that rely on the explicit representation of knowledge using formal languages—including formal logic—and the manipulation of language items (‘symbols’) by algorithms to achieve a goal. Mostly, neuro-symbolic AI utilizes formal logic as studied in the *knowledge representation and reasoning* subfield of AI, but the lines blur, and tasks such as general term rewriting or planning, that may not be framed explicitly in formal logic, bear significant similarities and should reasonably be included.

Two major reasons are usually brought forth to motivate the study of neuro-symbolic integration. The first one comes from the field of cognitive science, a highly interdisciplinary field that studies the human mind. In that context, we can understand artificial neural networks as an abstraction of the physical workings of the brain, while we can understand formal logic as an abstraction of what we perceive, through introspection, when contemplating explicit cognitive reasoning. In order to advance the understanding of the human mind, it therefore appears to be a natural question to ask how these two abstractions can be related or even unified, or how symbol manipulation can arise from a neural substrate [1].

The second reason is tied to the field of AI and is based on the observation that neural and symbolic approaches to AI complement each other with respect to their strengths and weaknesses. For example, deep learning systems are trainable from raw data and are robust against outliers or errors in the base data, while symbolic systems are brittle with respect to outliers and data errors, and are far less trainable. Symbolic systems, on the other hand, can make explicit use of expert knowledge, and are to a high extent self-explanatory, as their algorithms can be inspected and understood in detail by a human, while neural learning systems cannot readily take advantage of available coded expert knowledge, and are black boxes that make understanding their decision making processes very hard. It is therefore natural to ask how neural and symbolic approaches can be combined or even unified in order to overcome the weaknesses of either approach. Traditionally, in neuro-symbolic AI research, emphasis is on either incorporating symbolic abilities in a neural approach, or coupling neural and symbolic components such that they seamlessly interact [2].

Research in neuro-symbolic AI has a very long tradition, and we refer the interested reader to overview works such as Refs [1,3] that were written before the most recent developments. Indeed, neuro-symbolic AI has seen a significant increase in activity and research output in recent years, together with an apparent shift in emphasis, as discussed in Ref. [2]. Below, we identify what we believe are the main general research directions the field is currently pursuing. It is of course impossible to give credit to all nuances or all important recent contributions in such a brief overview, but we believe that our literature pointers provide excellent starting points for a deeper engagement with neuro-symbolic AI topics.

Like in so many other respects, deep learning has had a major impact on neuro-symbolic AI in recent years. This appears to manifest, on the one hand, in an almost exclusive emphasis on deep learning approaches as the neural substrate, while previous neuro-symbolic AI research often deviated from standard artificial neural network architectures [2]. On the other hand, the deep learning context appears to have led to a renewed realization of the importance of neuro-symbolic AI research, and consequently a significant increase in research papers, meetings and prominent public appearances of the topic [2], as well as discussion of the topic in public media [4]. This increase in activity is probably primarily due to the fact that advances in deep learning now make it possible to address challenge problems in neuro-symbolic AI that were quite out of reach before the advent of deep learning, thus adding to its attractivity for research and applications. However, we may also be seeing indications or a realization that pure deep-learning-based methods are likely going to be insufficient for certain types of problems that are now being investigated from a neuro-symbolic perspective.

While we cannot give the whole neuro-symbolic AI field due recognition in a brief overview, we have attempted to identify the major current research directions based on our survey of recent literature, and we present them below. Literature references within this text are limited to general overview articles, but a supplementary online document referenced at the end contains references to concrete examples from the recent literature. Examples for historic overview works that provide a perspective on the field, including cognitive science aspects, prior to the recent acceleration in activity, are Refs [1,3]. Very recent overview articles include Refs [2,5–9]. And recent article collections are, e.g. Refs [10–12].

**Solving symbolic problems with deep learning.** In this line of effort, deep learning systems are trained to solve problems such as term rewriting, planning, elementary algebra, logical deduction or abduction or rule learning. These problems are known to often require sophisticated and non-trivial symbolic algorithms. Attempting these hard but well-understood problems using deep learning adds to the general understanding of the capabilities and limits of deep learning. It also provides deep learning modules that are potentially faster (after training) and more robust to data imperfections than their symbolic counterparts.

**Using symbolic knowledge bases and expressive metadata to improve deep learning systems.** Metadata that augments network input is increasingly being used to improve deep learning system performances, e.g. for conversational agents. Metadata are a form of formally represented background knowledge, for example a knowledge base, a knowledge graph or other structured background knowledge, that adds further information or context to the data or system. In its simplest form, metadata can consist just of keywords, but they can also take the form of sizeable logical background theories. Neuro-symbolic lines of work include the use of knowledge graphs to improve zero-shot learning. Background knowledge can also be used to improve out-of-sample generalizability, or to ensure safety guarantees in neural control systems. Other work utilizes structured background knowledge for improving coherence and consistency in neural sequence models. In a similar manner, natural language fact statements can be used as background knowledge for deep-learning-based conversation agents—strictly speaking, we may not call this approach neuro-symbolic as it uses natural language rather than structured metadata, but of course work like this, using natural language, can be closely related.

**Explainability through background knowledge.** How to explain the input-output behavior, or even inner activation states, of deep learning networks is a highly important line of investigation, as the black-box character of existing systems hides system biases and generally fails to provide a rationale for decisions. Recently, awareness is growing that explanations should not only rely on raw system inputs but should reflect background knowledge.

**Complex problem solving through coupling of deep learning and symbolic components.** Coupled neuro-symbolic systems are increasingly used to solve complex problems such as game playing or scene, word, sentence interpretation. Coupled systems can also be used to make deep learning more sample efficient, for example by using symbolic planning to arrive at (among other things) more data-efficient reinforcement learning, or the use of coupling for vision and language understanding that also results in a more data- and memory-efficient approach. In a different line of work, logic tensor networks in particular have been designed to capture logical background knowledge to improve image interpretation, and neural theorem provers can provide natural language reasoning by also taking knowledge bases into account. Coupling may be through different methods, including the calling of deep learning systems within a symbolic algorithm, or the acquisition of symbolic rules during training. Very tight coupling can be achieved for example by means of Markov logics.

Summarizing, neuro-symbolic artificial intelligence is an emerging subfield of AI that promises to favorably combine knowledge representation and deep learning in order to improve deep learning and to explain outputs of deep-learning-based systems. Neuro-symbolic approaches carry the promise that they will be useful for addressing complex AI problems that cannot be solved by purely symbolic or neural means. We have laid out some of the most important currently investigated research directions, and provided literature pointers suitable as entry points to an in-depth study of the current state of the art.

## Supplementary Material

nwac035_Supplemental_File
