# Supplementary material for: Neuro-symbolic approaches in artificial intelligence
Source: Natl Sci Rev. 2022 Mar 4;9(6):nwac035. doi: 10.1093/nsr/nwac035 (PMC9166567; doi:10.1093/nsr/nwac035)
Supplement: nwac035_Supplemental_File [file nwac035_supplemental_file.pdf]

National Science Review, 2021, pp. 1–4

doi: DOI HERE
Advance Access Publication Date: Day Month Year

Supplementary References for: Neuro-Symbolic Approaches in Artificial Intelligence

Pascal Hitzler,[1,*] Aaron Eberhart,[1] Monireh Ebrahimi,[2] Md Kamruzzaman Sarker[3] and Lu Zhou[1]

[1]Department of Computer Science, Kansas State University, 1701D Platt St, Manhattan, 66506, Kansas, USA, [2]Center for Open Source Data and AI Technologies (CODAIT), IBM Watson, 425 Market St, San Francisco, 94105, California, USA and [3]Department of Computing Sciences, University of Hartford, 200 Bloomfield Avenue, West Hartford, 06117, Connecticut, USA

[*]Corresponding author. hitzler@ksu.edu

FOR PUBLISHER ONLY Received on Date Month Year; revised on Date Month Year; accepted on Date Month Year

Abstract

This supplementary document provides additional literature references for the article *Neuro-Symbolic Approaches in Artificial Intelligence* by Pascal Hitzler, Aaron Eberhart, Monireh Ebrahimi, Md Kamruzzaman Sarker, Lu Zhou, National Science Review. Table 1 shows which reference refers to which statement in the article.

Acknowledgement This work was partially supported by the National Science Foundation (NSF) under award OIA-2033521 *KnowWhereGraph: Enriching and Linking Cross-Domain Knowledge Graphs using Spatially-Explicit AI Technologies.*

References

1. Zeynep Akata, Dan Balliet, Maarten de Rijke, Frank Dignum, Virginia Dignum, Guszti Eiben, Antske Fokkens, Davide Grossi, Koen V. Hindriks, Holger H. Hoos, Hayley Hung, Catholijn M. Jonker, Christof Monz, Mark A. Neerincx, Frans A. Oliehoek, Henry Prakken, Stefan Schlobach, Linda C. van der Gaag, Frank van Harmelen, Herke van Hoof, Birna van Riemsdijk, Aimee van Wynsberghe, Rineke Verbrugge, Bart Verheij, Piek Vossen, and Max Welling. A research agenda for hybrid intelligence: Augmenting human intellect with collaborative, adaptive, responsible, and explainable artificial intelligence. *Computer*, 53(8):18–28, 2020.
2. Masataro Asai and Christian Muise. Learning neural-symbolic descriptive planning models via cube-space priors: The voyage home (to STRIPS). In Christian Bessiere, editor, *Proceedings of the Twenty-Ninth International Joint Conference on Artificial Intelligence, IJCAI 2020*, pages 2676–2682. ijcai.org, 2020.
3. Sebastian Bader and Pascal Hitzler. Dimensions of neural-symbolic integration – A structured survey. In Sergei N. Artëmov, Howard Barringer, Artur S. d'Avila Garcez, Luís C. Lamb, and John Woods, editors, *We Will Show Them! Essays in Honour of Dov Gabbay, Volume One*, pages 167–194. College Publications, 2005.
4. Tarek R. Besold, Artur d'Avila Garcez, Sebastian Bader, Howard Bowman, Pedro Domingos, Pascal Hitzler, Kai-Uwe Kühnberger, Luis C. Lamb, Priscila Machado Vieira Lima, Leo de Penning, Gadi Pinkas, Hoifung Poon, and Gerson Zaverucha. Neural-symbolic learning and reasoning: A survey and interpretation. In Pascal Hitzler and Md Kamruzzaman Sarker, editors, *Neuro-Symbolic Artificial Intelligence: The State of the Art*, Frontiers of Artificial Intelligence and Applications. IOS Press, 2021. To appear, available from `https://arxiv.org/abs/1711.03902`.
5. François Charton, Amaury Hayat, and Guillaume Lample. Learning advanced mathematical computations from examples. In *9th International Conference on Learning Representations, ICLR 2021, Virtual Event, Austria, May 3-7, 2021*. OpenReview.net, 2021.
6. Xinyun Chen and Yuandong Tian. Learning to perform local rewriting for combinatorial optimization. In Hanna M. Wallach, Hugo Larochelle, Alina Beygelzimer, Florence d'Alché-Buc, Emily B. Fox, and Roman Garnett, editors, *Advances in Neural*

© The Author 2021. Published by Oxford University Press. All rights reserved. For permissions, please e-mail: journals.permissions@oup.com

**Overviews and Collections**	
Recent overviews and collections	[7, 28, 21, 20, 1, 31, 24, 25, 8, 18]
Historic overviews and collections	[3, 16, 9, 4]
**Solving symbolic problems with deep learning**	
term rewriting	[6]
planning	[2]
elementary algebra	[5]
logical deduction	[13]
logical abduction	[40]
rule learning	[35, 14]
modules	[34]
**Using symbolic knowledge bases and expressive metadata to improve deep learning systems**	
conversational agents	[10]
metadata as logical background theories	[17]
improving zero-shot learning	[19]
safety guarantees in neural control systems	[37]
coherence and consistency in neural sequence models	[27]
natural language facts for conversation agents	[15]
**Explainable Artificial Intelligence**	
Explanations that reflect background knowledge	[33, 30, 38, 29]
**Complex problem solving through coupling of deep learning and symbolic components**	
game playing	[32]
scene, word, sentence interpretation	[23]
more data-efficient reinforcement learning	[36]
vision and language understanding	[39]
image interpretation	[12]
natural language reasoning	[26]
coupling by calling	[22]
rule acquisition during training	[5, 12]
Markov Logic	[11]

Table 1. Supplementary references and how they relate to the statements in the main article.

Information Processing Systems 32: Annual Conference on Neural Information Processing Systems 2019, NeurIPS 2019, December 8-14, 2019, Vancouver, BC, Canada, pages 6278–6289, 2019.

7. Artur S. d'Avila Garcez, Marco Gori, Luís C. Lamb, Luciano Serafini, Michael Spranger, and Son N. Tran. Neural-symbolic computing: An effective methodology for principled integration of machine learning and reasoning. *IfCoLog Journal of Logics and their Applications*, 6(4):611–632, 2019.
8. Artur S. d'Avila Garcez and Ernesto Jiménez-Ruiz, editors. *Proceedings of the 15th International Workshop on Neural-Symbolic Learning and Reasoning as part of the 1st International Joint Conference on Learning & Reasoning (IJCLR 2021), Virtual conference, October 25-27, 2021*, volume 2986 of *CEUR Workshop Proceedings*. CEUR-WS.org, 2021.
9. Artur S. d'Avila Garcez, Luís C. Lamb, and Dov M. Gabbay. *Neural-Symbolic Cognitive Reasoning*. Cognitive Technologies. Springer, 2009.
10. Emily Dinan, Stephen Roller, Kurt Shuster, Angela Fan, Michael Auli, and Jason Weston. Wizard of Wikipedia: Knowledge-powered conversational agents. In *7th International Conference on Learning Representations, ICLR 2019, New Orleans, LA, USA, May 6-9, 2019*. OpenReview.net, 2019.
11. Pedro M. Domingos and Daniel Lowd. Unifying logical and statistical AI with markov logic. *Commun. ACM*, 62(7):74–83, 2019.
12. Ivan Donadello, Luciano Serafini, and Artur S. d'Avila Garcez. Logic tensor networks for semantic image interpretation. In Carles Sierra, editor, *Proceedings of the Twenty-Sixth International Joint Conference on Artificial Intelligence, IJCAI 2017, Melbourne, Australia, August 19-25, 2017*, pages 1596–1602. ijcai.org, 2017.
13. Monireh Ebrahimi, Aaron Eberhart, Federico Bianchi, and Pascal Hitzler. Towards bridging the neuro-symbolic gap: deep deductive reasoners. *Applied Intelligence*, 51(9):6326–6348, 2021.
14. Richard Evans and Edward Grefenstette. Learning explanatory rules from noisy data. *J. Artif. Intell. Res.*, 61:1–64, 2018.
15. Marjan Ghazvininejad, Chris Brockett, Ming-Wei Chang, Bill Dolan, Jianfeng Gao, Wen-tau Yih, and Michel Galley. A knowledge-grounded neural conversation model. In Sheila A. McIlraith and Kilian Q. Weinberger, editors, *Proceedings of the Thirty-Second AAAI Conference on Artificial Intelligence, (AAAI-18), the 30th innovative Applications of Artificial Intelligence (IAAI-18), and the 8th AAAI Symposium on Educational Advances in Artificial Intelligence (EAAI-18), New Orleans, Louisiana, USA, February 2-7, 2018*, pages 5110–5117. AAAI Press, 2018.

16. Barbara Hammer and Pascal Hitzler, editors. *Perspectives of Neural-Symbolic Integration*, volume 77 of *Studies in Computational Intelligence*. Springer, 2007.
17. Pascal Hitzler. A review of the semantic web field. *Commun. ACM*, 64(2):76–83, 2021.
18. Pascal Hitzler and Md Kamruzzaman Sarker, editors. *Neuro-Symbolic Artificial Intelligence: The State of the Art*. Frontiers in Artificial Intelligence and Applications. IOS Press, 2022. To appear.
19. Michael Kampffmeyer, Yinbo Chen, Xiaodan Liang, Hao Wang, Yujia Zhang, and Eric P. Xing. Rethinking knowledge graph propagation for zero-shot learning. In *IEEE Conference on Computer Vision and Pattern Recognition, CVPR 2019, Long Beach, CA, USA, June 16-20, 2019*, pages 11487–11496. Computer Vision Foundation / IEEE, 2019.
20. Stefan Kramer. A brief history of learning symbolic higher-level representations from data (and a curious look forward). In Christian Bessiere, editor, *Proceedings of the Twenty-Ninth International Joint Conference on Artificial Intelligence, IJCAI 2020*, pages 4868–4876. ijcai.org, 2020.
21. Luís C. Lamb, Artur S. d'Avila Garcez, Marco Gori, Marcelo O. R. Prates, Pedro H. C. Avelar, and Moshe Y. Vardi. Graph neural networks meet neural-symbolic computing: A survey and perspective. In Christian Bessiere, editor, *Proceedings of the Twenty-Ninth International Joint Conference on Artificial Intelligence, IJCAI 2020*, pages 4877–4884. ijcai.org, 2020.
22. Robin Manhaeve, Sebastijan Dumancic, Angelika Kimmig, Thomas Demeester, and Luc De Raedt. Neural probabilistic logic programming in DeepProbLog. *Artif. Intell.*, 298:103504, 2021.
23. Jiayuan Mao, Chuang Gan, Pushmeet Kohli, Joshua B. Tenenbaum, and Jiajun Wu. The neuro-symbolic concept learner: Interpreting scenes, words, and sentences from natural supervision. In *7th International Conference on Learning Representations, ICLR 2019, New Orleans, LA, USA, May 6-9, 2019*. OpenReview.net, 2019.
24. Gary Marcus and Ernest Davis. Insights for AI from the human mind. *Commun. ACM*, 64(1):38–41, 2021.
25. Andreas Martin, Knut Hinkelmann, Hans-Georg Fill, Aurona Gerber, Doug Lenat, Reinhard Stolle, and Frank van Harmelen, editors. *Proceedings of the AAAI 2021 Spring Symposium on Combining Machine Learning and Knowledge Engineering (AAAI-MAKE 2021), Stanford University, Palo Alto, California, USA, March 22-24, 2021*, volume 2846 of *CEUR Workshop Proceedings*. CEUR-WS.org, 2021.
26. Pasquale Minervini, Matko Bosnjak, Tim Rocktäschel, Sebastian Riedel, and Edward Grefenstette. Differentiable reasoning on large knowledge bases and natural language. In Ilaria Tiddi, Freddy Lécué, and Pascal Hitzler, editors, *Knowledge Graphs for eXplainable Artificial Intelligence: Foundations, Applications and Challenges*, volume 47 of *Studies on the Semantic Web*, pages 125–142. IOS Press, 2020.
27. Maxwell I. Nye, Michael Henry Tessler, Joshua B. Tenenbaum, and Brenden M. Lake. Improving coherence and consistency in neural sequence models with dual-system, neuro-symbolic reasoning. *CoRR*, abs/2107.02794, 2021.
28. Luc De Raedt, Sebastijan Dumancic, Robin Manhaeve, and Giuseppe Marra. From statistical relational to neuro-symbolic artificial intelligence. In Christian Bessiere, editor, *Proceedings of the Twenty-Ninth International Joint Conference on Artificial Intelligence, IJCAI 2020*, pages 4943–4950. ijcai.org, 2020.
29. Natalia Díaz Rodríguez, Alberto Lamas, Jules Sanchez, Gianni Franchi, Ivan Donadello, Siham Tabik, David Filliat, Policarpo Cruz, Rosana Montes, and Francisco Herrera. EXplainable Neural-Symbolic Learning (X-NeSyL) methodology to fuse deep learning representations with expert knowledge graphs: the MonuMAI cultural heritage use case. *Information Fusion*, 2021. To appear.
30. Md. Kamruzzaman Sarker, Joshua Schwartz, Pascal Hitzler, Lu Zhou, Srikanth Nadella, Brandon S. Minnery, Ion Juvina, Michael L. Raymer, and William R. Aue. Wikipedia knowledge graph for explainable AI. In Boris Villazón-Terrazas, Fernando Ortiz-Rodríguez, Sanju Mishra Tiwari, and Shishir K. Shandilya, editors, *Knowledge Graphs and Semantic Web – Second Iberoamerican Conference and First Indo-American Conference, KGSWC 2020, Mérida, Mexico, November 26-27, 2020, Proceedings*, volume 1232 of *Communications in Computer and Information Science*, pages 72–87. Springer, 2020.
31. Md Kamruzzaman Sarker, Lu Zhou, Aaron Eberhart, and Pascal Hitzler. Neuro-symbolic artificial intelligence: Current trends. *AI Communications*, 2021. Accepted with minor revisions, available from `https://arxiv.org/abs/2105.05330`.
32. David Silver, Julian Schrittwieser, Karen Simonyan, Ioannis Antonoglou, Aja Huang, Arthur Guez, Thomas Hubert, Lucas Baker, Matthew Lai, Adrian Bolton, Yutian Chen, Timothy P. Lillicrap, Fan Hui, Laurent Sifre, George van den Driessche, Thore Graepel, and Demis Hassabis. Mastering the game of Go without human knowledge. *Nat.*, 550(7676):354–359, 2017.
33. Ilaria Tiddi, Freddy Lécué, and Pascal Hitzler, editors. *Knowledge Graphs for eXplainable Artificial Intelligence: Foundations, Applications and Challenges*, volume 47 of *Studies on the Semantic Web*. IOS Press, 2020.
34. Michael van Bekkum, Maaike de Boer, Frank van Harmelen, André Meyer-Vitali, and Annette ten Teije. Modular design patterns for hybrid learning and reasoning systems. *Appl. Intell.*, 51(9):6528–6546, 2021.
35. Wenhan Xiong, Thien Hoang, and William Yang Wang. Deeppath: A reinforcement learning method for knowledge graph reasoning. In Martha Palmer, Rebecca Hwa, and Sebastian Riedel, editors, *Proceedings of the 2017 Conference on Empirical Methods in Natural Language Processing, EMNLP 2017, Copenhagen, Denmark, September 9-11, 2017*, pages 564–573. Association for Computational Linguistics, 2017.
36. Fangkai Yang, Daoming Lyu, Bo Liu, and Steven Gustafson. PEORL: integrating symbolic planning and hierarchical reinforcement learning for robust decision-making. In Jérôme Lang, editor, *Proceedings of the Twenty-Seventh International Joint Conference on Artificial Intelligence, IJCAI 2018, July 13-19, 2018, Stockholm, Sweden*, pages 4860–4866. ijcai.org, 2018.
37. Pengfei Yang, Jianlin Li, Jiangchao Liu, Cheng-Chao Huang, Renjue Li, Liqian Chen, Xiaowei Huang, and Lijun Zhang. Enhancing robustness verification for deep neural networks via symbolic propagation. *Formal Aspects Comput.*, 33(3):407–435, 2021.
38. Chih-Kuan Yeh, Been Kim, Sercan Ömer Arik, Chun-Liang Li, Tomas Pfister, and Pradeep Ravikumar. On completeness-aware concept-based explanations in deep neural networks. In Hugo Larochelle, Marc'Aurelio Ranzato, Raia Hadsell, Maria-Florina

Balcan, and Hsuan-Tien Lin, editors, *Advances in Neural Information Processing Systems 33: Annual Conference on Neural Information Processing Systems 2020, NeurIPS 2020, December 6-12, 2020, virtual*, 2020.

39. Kexin Yi, Jiajun Wu, Chuang Gan, Antonio Torralba, Pushmeet Kohli, and Josh Tenenbaum. Neural-symbolic VQA: disentangling reasoning from vision and language understanding. In Samy Bengio, Hanna M. Wallach, Hugo Larochelle, Kristen Grauman, Nicolò Cesa-Bianchi, and Roman Garnett, editors, *Advances in Neural Information Processing Systems 31: Annual Conference on Neural Information Processing Systems 2018, NeurIPS 2018, December 3-8, 2018, Montréal, Canada*, pages 1039–1050, 2018.
40. Zhi-Hua Zhou. Abductive learning: towards bridging machine learning and logical reasoning. *Sci. China Inf. Sci.*, 62(7):76101:1–76101:3, 2019.
